# Gender-role behaviour and gender identity in girls with classical congenital adrenal hyperplasia

**DOI:** 10.1186/s12887-021-02742-9

**Published:** 2021-06-05

**Authors:** Sumudu Nimali Seneviratne, Umesh Jayarajah, Shamaali Gunawardana, Malik Samarasinghe, Shamya de Silva

**Affiliations:** 1 Department of Pediatrics, Faculty of Medicine, University of Colombo, Colombo, Sri Lanka; 2 Department of Surgery, Faculty of Medicine, University of Colombo, Colombo, Sri Lanka

**Keywords:** Gender identity questionnaire for children (GIQC), South Asia, Sri Lanka, 21-hydroxylase deficiency, Lower middle income countries (LMIC)

## Abstract

**Introduction:**

Girls with classical congenital adrenal hyperplasia (CAH) are exposed to excess fetal adrenal androgens in-utero, and often born with masculinised genitalia. They are conventionally reared as females, but show more “boyish” gender-role behaviour (GRB) and gender-identity (GI) issues in childhood and adolescence. Male-rearing is also reported mainly due to delayed treatment and/or socio-cultural factors. We compared GRB/GI in girls with CAH with healthy age matched children, and explored for associations with socio-demographic and diagnosis/treatment related factors.

**Methods:**

GRB and GI were assessed using the Gender Identity Questionnaire for children (GIQC) in 27 girls with classical CAH at a specialised clinic, and compared with 50 age-matched healthy controls, with exploratory-analysis based on socio-demographic and diagnosis/treatment-related factors.

**Results:**

Girls with CAH had lower total GIQC scores compared to healthy children (3.29 vs. 4.04, *p* = < 0.001) with lower GRB score (3.39 vs. 4.23, *p* < 0.001), and tendency for lower GI score (3.19 vs. 3.5, *p* = 0.08). Exploratory analysis showed no differences based on diagnosis/treatment factors including age, degree of virilisation at diagnosis and surgical procedures. and only subtle changes based on ethnicity and maternal education.

**Discussion/conclusion:**

Girls with CAH managed at a specialised centre showed more masculinised GRB and tendency for ambiguous GI, which did not vary upon diagnosis/treatment related factors, suggesting that prenatal androgen exposure was the likely contributor. Clinicians should be vigilant about the increased risk of gender-related problems in girls with CAH, irrespective of sociocultural background and despite early treatment.

**Supplementary Information:**

The online version contains supplementary material available at 10.1186/s12887-021-02742-9.

## Introduction

Classical congenital adrenal hyperplasia (C-CAH) is the commonest disorder/ difference of sex development (DSD) under the 46, XX DSD subgroup. Females with classical CAH often have virilised genitalia at birth due to excessive fetal adrenal androgen production, but are conventionally reared as girls, mainly due to future female reproductive potential [1, 2]. However, it is postulated that in-utero “virilisation” of the brain may also occur, which can affect gender-role behaviour (GRB) and gender-identity (GI) in childhood and adulthood [3]. Western studies report that girls with CAH show more masculinised behaviour in childhood, but mostly retain their female gender identity [1, 4–8], while about 5% of females with CAH have gender dysphoria/ undergo gender reassignment to male.

Gender-role behaviour (GRB) refers to behavioural characteristics associated with being male or female, while gender identity (GI) refers to the sense of self as male or female, or neither [9]. Children demonstrate GRB and GI through play, playmate selection, and expressed ideas about gender and satisfaction with assigned gender [10]. GRB and GI develop gradually in childhood, and is influenced by early life exposure to sex hormones, and social upbringing i.e., modelling and reinforcement of gender typical behaviour [3, 9, 11, 12]. Children and adolescents with atypical gender identity may experience psychological distress and social stigmatization [13, 14]. There are a few standardised tools designed to measure GRB and GI in children, including the parent-reported Gender Identity Questionnaire for Children (GIQC), which has excellent psychometric properties, has been used previously to study children with CAH, and is a useful screening tool for clinical settings [11, 15, 16] .

Gender-related issues in CAH are complex. Socio-cultural background, and delayed diagnosis/ inadequate treatment, can influence GRB/GI in girls with CAH [2, 17]. Data is available however, mainly from high-income countries in Europe and North America and there is a lack of data from Low-Middle Income Countries (LMIC), where socio-cultural background and medical care will differ. Further, male gender assignment in girls with CAH appears to be more frequent in LMICs, in children with delayed diagnosis and/or higher degree of virilisation [1–3, 18]. Sri Lanka is a LMIC in South Asia, with a multi-ethnic population, and free access to health care and education. Children throughout the country have access to specialised Pediatric endocrine services at the Lady Ridgeway Hospital (LRH), Colombo, the main tertiary-care children’s hospital in Sri Lanka. However, neonatal screening for CAH is not performed in Sri Lanka. In this study, we assessed GRB and GI in Sri Lankan girls with CAH, and hypothesized that they would show lower conformity to gender of rearing, compared to healthy age-matched children without CAH/DSD. Further, we also hypothesized that sociodemographic factors influencing gender-related upbringing (ethnicity, parental education, income level, age) could cause variation in gender identity, while children with more severe disease and/or suboptimal disease control (SW-CAH, delayed diagnosis, higher Prader stage, need for surgery, gender reassignment, and precocious puberty) would have more gender incongruity. Thus, we explored associations between GRB/GI and socio-demographic and disease/treatment related factors.

## Materials and methods

This observational study was conducted ethically in accordance with the Declaration of Helsinki. Ethical approval was obtained from the Ethics Review Committee of Faculty of Medicine, University of Colombo. Parents of participating children provided written informed consent. The study was conducted between 2017 and 2019, including all children with classical CAH above 2.5 years of age reared as girls, followed up at the University Pediatric Endocrine clinic of LRH, Colombo. The diagnosis of CAH was made clinically and biochemically. Basic socio-demographic details were collected from all participants, and diagnosis/treatment factors were obtained from clinical records. Fifty age-matched healthy children without CAH/DSDs, or serious chronic illness attending a General Pediatric clinic were included as controls. We included both girls and boys without CAH/DSD in the control group, as we wanted to compare deviation of gender behaviour in girls with CAH in comparison to healthy children (both girls and boys) without CAH/ DSD, considering gender as a spectrum, rather than a two-ended entity.

Gender identity was assessed by the Parent-report GIQC, a pre-validated standardised instrument developed to detect cross gender identity in children above 2.5 years of age [19]. It utilizes a 5-point Likert response scale, and consists of 16-items related to core phenomenology of gender identity with first 12-items pertaining to GRB (gender-related play, playmate preferences), and last 4-items to GI (identification, like/dislike towards assigned sex, anatomic dysphoria) [11, 15, 20]. The total GIQC score was obtained by averaging the scores from all completed questions; GRB sub score by averaging questions 1–12; and GI sub score by averaging questions 13–16. Higher scores indicated better conformity to gender of rearing. A total GIQC score of 3 or less was taken as indicative of gender dysphoria [19]. The GIQC was translated and contextually adapted according to WHO guidelines of translation and adaptation of instruments. It was forward translated by two independent translators to produce a single reconciled version and back translated and compared with the source instruments to make amendments. The questionnaire was then pre-tested, and modified to be culturally appropriate under supervision of context experts and administered to mothers of all participants [21].

### Statistical analysis

Categorical variables were expressed as frequencies and percentages and continuous variables as mean, median and range, with no imputation for missing data. Comparisons between cases and controls, including subgroup analyses based on age range, ethnicity and parental education, and exploratory analysis in girls with CAH based on socioeconomic and diagnosis/treatment related factors were performed using non-parametric tests: Chi-square test for categorical data and Mann Whitney-U test for continuous variables using SPSS statistical software ver.21. Level of significance was set at *p* < 0.05.

## Results

The study group consisted of 27 girls with C-CAH (mean age: 9.8 range 3–16 years). The control group consisted of 25 girls (mean age: 9.2, range: 3–15 years) and 25 boys (mean age: 9.7, range: 4–15 years) without CAH/DSD. Mothers and fathers of all study participants had completed primary education, and more than a third of mothers in both groups had completed secondary education/ received tertiary education.

The study group included 18 girls with SW-CAH (Salt Wasting CAH) and 9 girls with SV-CAH (Simple Virilizing CAH). A substantial number of patients (43.5%) were from low income families (monthly household net income ≤ LKR 25,000 (approximately 108 Euros). The mean age at diagnosis was 1.2 years (range; 1 day to 12 years) Presentation was delayed beyond 2 years of age in 21%. Genital virilisation was Prader stage 4/5 at presentation in more than half the girls (54%). Two were registered as boys at birth, and reassigned to female gender at 10 months and 4 years of age. Four experienced central precocious puberty, which was suppressed by GnRH analogues. A majority (81%) had undergone one or more feminising genital surgeries, with first surgery performed at a mean age of 4.4 years (range: 1.2–10.7), while average time elapsed after surgery was 4.5 years (range: 0.55–9.9).

The mean GIQC score in girls with CAH was 3.29 (median: 3.31, range: 1.81–4.13); mean subscale score for GRB was 3.39 (range: 2–4.33), and for GI, 3.2 (range: 1.5–5.0). The mean GIQC score in girls with CAH (3.29 vs. 4.04, *p* < 0.001) as well as the mean GRB sub score were significantly lower than that of the control group (3.39 vs. 4.23, *p* < 0.001), while the mean GI sub scores in girls with CAH showed only a tendency to be lower (3.19 vs. 3.5, *p* = 0.08). Scores suggestive of gender dysphoria were seen in 6 girls with CAH (22%), and none within the control group.

In subgroup analysis, GIQC scores in girls with CAH remained significantly lower than in controls: in all age groups; all ethnic groups; and irrespective of parental educational levels (Table 1). Sub scores for GRB showed a pattern similar to total GIQC scores, except for lack of significant differences in Muslim ethnicity, and lower maternal education group. In contrast, GI sub scores which only showed a tendency to differ in the overall cohort, were significantly lower in CAH girls of Sinhalese ethnicity, compared to controls (*p* = 0.010) (Supplementary Table 1).

**Table 1:** Median GIQC score between study and control groups based on age, ethnicity and parental educational level

Demographic factors		Cases (*n* = 27)		Controls (*n* = 50)		*p*-value
		n	Median GIQC	n	Median GIQC	
Age	2.5–6.9	6	3.31	13	4.25	< 0.001**
	7–11.9	14	3.50	26	4.00	0.001**
	> 12	7	2.87	11	4.13	0.002**
Ethnicity	Sinhalese (S)	16	3.30	28	4.00	< 0.001**
	Tamil(T)	5	3.13	11	4.13	0.003**
	Muslim (M)	6	3.72	11	4.07	0.02*
Maternal education	Not completed	11	3.28	18	4.00	0.02*
	Completed school or higher	16	3.56	32	4.06	0.001**
Paternal education	Not completed	7	3.30	18	4.00	< 0.001**
	Completed school or higher	20	3.56	32	4.07	0.004**

*GIQC* Gender Identity Questionnaire for Children

*Statistically significant at *p* = 0.05 level, **Statistically significant at *p* = 0.01 level

On exploratory analysis, among girls with CAH, we found no significant difference based on diagnosis/treatment related factors in GIQC scores (Table 2) or GRB/GI sub scores (Supplementary Table 2) Further, there were no differences in total GIQC scores among girls with CAH based on socio-economic factors (Table 2). On GRB and GI sub score exploration however, girls with CAH from Sinhalese ethnicity had significantly lower GI scores compared to those from other ethnicities (2.88 vs. 3.5, *p* = 0.013) (Supplementary Table 2).

**Table 2:** Differences in GIQC score among girls with CAH based on diagnosis/treatment related factors and socio-demographic factors

Variable	Category	n	GIQC Score		*p*-value
			Median	Range	
Type of CAH	Salt wasting	18	3.5	2.3–4.1	0.339
	Non salt wasting	9	3.3	1.8–4.0	
Age at diagnosis	< 2 years	19	3.3	2.3–4.1	0.746
	≥2 years	4	3.5	1.8–4.0	
Prader stage at diagnosis	4,5	13	3.3	2.3–4.1	0.954
	1,2,3	11	3.2	1.8–3.8	
Underwent surgery	Yes	22	3.3	2.3–4.1	0.212
	No	5	3.8	1.8–4.0	
Reassignment of gender	Yes	2	3.1	3.0–3.3	0.331
	No	25	3.4	1.8–4.1	
Precocious puberty	Present	4	3.6	2.3–4.1	0.465
	Absent	19	3.3	1.8–3.9	
Mother’s Education: completed up to advanced level at school or higher	No	16	3.3	2.3–4.0	0.505
	Yes	11	3.6	1.8–4.1	
Father’s Education: completed up to advanced level at school or higher	No	20	3.3	1.8–4.0	0.376
	Yes	7	3.6	3.0–4.1	
Monthly income of the family (Rs.)	≤25,000	10	3.2	2.3–3.8	0.172
	> 25,000	13	3.6	1.8–4.1	
Ethnicity	Sinhalese	16	3.3	1.8–4.1	0.401
	Other	11	3.5	2.3–4.0	
Age at assessment	≤12 years	20	3.4	2.3–4.1	0.288
	> 12 years	7	3.0	1.8–4.0	

*GIQC* Gender Identitiy Questionnaire for children, *CAH* Congenital Adrenal Hyperplasia

## Discussion/conclusion

In this study, Sri Lankan girls with CAH showed lower GIQC score (mean 3.3) compared to age-matched healthy controls, and more than a fifth of girls with CAH (22%) had scores suggestive of gender dysphoria indicating less conformity with gender of rearing in keeping with our hypothesis. These differences were more convincing for gender-role behaviour, and less for gender identity. However, neither sociodemographic factors, nor diagnosis/ treatment related factors we assessed appeared to influence GIQC scores significantly, suggesting that the major reason for gender incongruence was prenatal exposure to excess androgens rather than socio-cultural upbringing or postnatal management. Interestingly, GI issues appeared to be significant in girls with CAH of Sinhalese ethnicity, for which the reasons were not apparent from this study.

These study findings support previous research from other regions of the world. When considering high income countries, a multicentre-study on gender development from the UK, similarly reported lower GIQC scores in 43 girls with classical CAH compared to healthy controls, with greater deviation in GRB sub score than GI sub score [11]. In Australia, 19 girls with 46XX DSD (including 16 with CAH), had a mean GIQC score of 3.6, with 16% having scores indicating gender dysphoria [19]. Our study findings are compatible with these studies, with perhaps more gender incongruence. Dresens et al who combined outcomes data on gender development, in 250 girls and women with CAH from North America and Western Europe, reported only 13 (5%) had features of gender dysphoria [1].

There appears to be greater tendency towards male gender of rearing reported from small studies from LMIC in Asia [12, 20, 21]. Fifteen Indonesian girls with SV CAH had lower GIQC scores compared to age matched control girls, and three who were untreated had converted to male gender by 2–3 years of age [20]. Among eleven Bangladeshi girls with CAH, 4 (36%) had gender identity scores compatible with male gender, while another 3 with untreated SV CAH had converted to male gender in early childhood [21]. In Indian girls with CAH being reared as female, 1/13 (8%) had a low GIQC score indicating gender dysphoria, while two females being raised as males, showed good conformity to male gender [12]. During our study, we found only one child with CAH (1/28, 4%) being reared as a boy. Thus, our study appears to be one of the larger studies in girls with CAH from LMIC in Asia, and our findings appear to lie somewhat in-between data from developed nations, and LMIC countries mentioned above, possibly reflecting the fact that all girls with CAH in our study had free access to specialised Pediatric care and relatively early treatment.

There is a paucity of data on the influence of socio-cultural factors on gender development in CAH [2]. A recent review reporting gender dysphoria rates among girls with CAH patients found wide variation (6.3 to 27.2%) across different settings, and highlighted the need to explore hereto unexplored cultural biases, and factors such as illiteracy, religious dogma and unavailability of specialized health care providers which may play a significant role in the observed variations [2]. Higher rates of male rearing in genetic females with CAH have been reported in cultures strongly favouring males on religious, financial or societal grounds, and patients from underdeveloped countries with late diagnosis and non-accessibility to the correct treatment. When exploring the influence of socio-demographic factors, we did not find significant differences in GIQC scores based on age, ethnicity, and parental education level, but did observe subtle variations in GI and GRB sub scores based on ethnicity, and maternal education. These variations could perhaps be explained by some of the factors above, while universal access to free education and specialised health care could have helped mitigate marked variations. CAH appears to be less prevalent among those of Sinhalese ethnicity compared to ethnic minority groups in Sri Lanka [22]. We speculate that greater gender identity issues identified among Sinhalese girls could perhaps be a consequence of it being less common, but also propose that it is more likely that the underlying reasons could be more complex, and influenced by complex social interactions between ethnicity, religion and social class, which will require in-depth study to unravel [23]. Gender non-conformity is related to behavioural and emotional challenges, especially in the setting of parents/guardians who endorse gender-stereotyped attitudes, and psychological counselling should be provided to such families, as it can help improve the social environment and thus improve psychological well-being among gender-nonconforming children [24, 25].

Non-heterosexual orientation, and masculinised behaviour have been reported in females with CAH probably due to high androgen exposure during fetal life, with some studies also showning a correlation with the severity of the phenotype/genotype [7, 26]. While it is plausible that girls with more intense prenatal exposure to androgens, (SW phenotypes with null genotypes, Prader 4/5 virilisation) as well as prolonged postnatal exposure to androgens (i.e., late diagnosed SV-CAH and those without access to early treatment) could have more severe gender related issues, this has not been proven by research findings to date [2]. We too found no differences in gender-related issues among girls with CAH based on: type of CAH; age and degree of virilisation at diagnosis; and undergoing gender reassignment, central precocious puberty, or feminizing surgery. Previous studies have reported similar results, except in cases with very delayed diagnosis/treatment [2, 27]. Berenbanum et al reported that gender identity in American girls with CAH was not related to degree of genital virilization, age at diagnosis, salt-wasting status, and age at genital reconstructive surgery [28]. Ediati et al reported that Indonesian adolescents and adult females with SV CAH, who had never received treatment, had significantly more gender dysphoria, than those who had received some glucocorticoid treatment [20]. Our cohort of children with CAH had continuous long-term access to glucocorticoids and fludrocortisone, which may have helped avoid worsening of gender-related identity issues, noted in some other LMICs.

One of our study limitations is the study setting being a tertiary care specialised clinic. While this could potentially have resulted in under-representation of those with more delayed diagnosis, we do not believe this to be the case, as children with CAH from all parts of the country including underprivileged rural areas, are referred to our centre. Furthermore, most studies done elsewhere have also been conducted in specialised care settings, increasing comparability. Further, it should be noted that we conducted multiple analysis for secondary outcomes based on socioeconomic and disease/treatment related factors in a relatively modest study sample, which could have increased the likelihood of type 1 error. These secondary outcomes are thus of an exploratory nature, and require further studies, preferably by mixed-methods research methodology prior to confirmation.

In conclusion, we found significant gender-related issues among girls with CAH in this study compared to healthy controls. Thus, we suggest that clinicians managing girls with CAH should screen for gender-related problems using specific questionnaires such as the GIQC, and provide psychological counselling for families of children/adolescents with significant gender non-conformity when possible [24, 25].

## Supplementary Information

**Additional file 1: Supplementary table 1.** Median GRB and GI scores between study and control groups based on age, ethnicity and parental educational level.

**Additional file 2: Supplementary table 2.** Differences in GRB and GI scores among girls with CAH based on diagnosis/treatment related factors and socio-demographic factors.

## Abbreviations

CAH: Congenital Adrenal Hyperplasia
DSD: Disorder/difference of Sex Development
GRB: Gender Related Behaviour
GI: Gender Identity
GIQC: Gender Identity Questionnaire for Children
LMIC: Low Middle Income Countries
SW: Salt Wasting
SV: Simple Virilizing
