# Supplementary material for: Gender-role behaviour and gender identity in girls with classical congenital adrenal hyperplasia
Source: BMC Pediatr. 2021 Jun 5;21:262. doi: 10.1186/s12887-021-02742-9 (PMC8178869; doi:10.1186/s12887-021-02742-9)
Supplement: Supplementary file 1 — Additional file 1: Supplementary table 1. Median GRB and GI scores between study and control groups based on age, ethnicity and parental educational level. [file 12887_2021_2742_MOESM1_ESM.docx]

Supplementary table 1: Median GRB and GI scores between study and control groups based on age, ethnicity and parental educational level

| Demographic factors | | N  Cases/ Controls | Median GRB | | *p*-value | Median GI | | *p*-value |
| --- | --- | --- | --- | --- | --- | --- | --- | --- |
|  |  |  | Cases (n=27) | Controls (n=50) |  | Cases (n=27) | Controls (n=50) |  |
| Age | 2.5-6.9 | 6/13 | 3.50 | 4.33 | <0.001** | 3.25 | 3.50 | 0.536 |
|  | 7-11.9 | 14/26 | 3.67 | 4.20 | 0.001** | 3.38 | 3.75 | 0.424 |
|  | >12 | 7/11 | 2.95 | 4.55 | 0.003** | 2.63 | 4.00 | 0.180 |
| Ethnicity | Sinhalese (S) | 16/28 | 3.48 | 4.25 | <0.001** | 2.88 | 3.75 | 0.010** |
|  | Tamil(T) | 5/11 | 3.00 | 4.33 | 0.005** | 3.50 | 4.00 | 0.743 |
|  | Muslim (M) | 6/11 | 3.67 | 4.18 | 0.122 | 3.50 | 3.50 | 0.884 |
| Maternal education | Not completed | 11/18 | 3.18 | 4.19 | 0.122 | 3.38 | 3.50 | 0.884 |
|  | Completed school or higher | 16/32 | 3.70 | 4.29 | 0.001** | 3.00 | 4.00 | 0.061 |
| Paternal education | Not completed | 7/18 | 3.33 | 4.19 | <0.001** | 3.13 | 3.50 | 0.238 |
|  | Completed school or higher | 20/32 | 3.67 | 4.33 | 0.001** | 3.25 | 4.00 | 0.220 |

** Statistically significant at p=0.01 level

GRB, Gender Role Behaviour; GI, Gender Identity
