# Supplementary material for: Gender-role behaviour and gender identity in girls with classical congenital adrenal hyperplasia
Source: BMC Pediatr. 2021 Jun 5;21:262. doi: 10.1186/s12887-021-02742-9 (PMC8178869; doi:10.1186/s12887-021-02742-9)
Supplement: Supplementary file 2 — Additional file 2: Supplementary table 2. Differences in GRB and GI scores among girls with CAH based on diagnosis/treatment related factors and socio-demographic factors. [file 12887_2021_2742_MOESM2_ESM.docx]

Supplementary table 2: Differences in GRB and GI scores among girls with CAH based on diagnosis/treatment related factors and socio-demographic factors

| Variable | Category | n | GRB | | *p*-value | GI | | *p*-value |
| --- | --- | --- | --- | --- | --- | --- | --- | --- |
|  |  |  | Median | Range |  | Median | Range |  |
| Type of CAH | Salt wasting | 18 | 3.67 | 2.45 - 4.25 | 0.298 | 3.25 | 1.50 - 5.00 | 0.980 |
|  | Non salt wasting | 9 | 3.30 | 2.00 - 4.33 |  | 3.00 | 2.25 - 5.00 |  |
| Age at diagnosis | <2years | 19 | 3.42 | 2.00 - 4.18 | 0.505 | 3.50 | 2.00 - 5.00 | 0.250 |
|  | ≥2 years | 4 | 3.60 | 2.50 - 4.33 |  | 2.88 | 2.25 - 3.50 |  |
| Prader stage at diagnosis | 4,5 | 13 | 3.30 | 2.45 - 4.25 | 0.865 | 3.25 | 1.50 - 4.00 | 0.910 |
|  | 1,2,3 | 11 | 3.50 | 2.00 - 4.10 |  | 3.00 | 2.00 - 5.00 |  |
| Underwent surgery | Yes | 22 | 3.38 | 2.00 - 4.25 | 0.232 | 3.25 | 1.50 - 5.00 | 0.739 |
|  | No | 5 | 4.10 | 2.50 - 4.33 |  | 3.25 | 2.25 - 5.00 |  |
| Reassignment of gender | Yes | 2 | 3.15 | 3.00 - 3.30 | 0.513 | 3.13 | 2.25 - 4.00 | 0.963 |
|  | No | 25 | 3.50 | 2.00 - 4.33 |  | 3.25 | 1.50 - 5.00 |  |
| Precocious puberty | Present | 4 | 3.59 | 2.00 - 4.33 | 0.725 | 3.50 | 3.00 - 4.00 | 0.611 |
|  | Absent | 19 | 3.45 | 2.45 - 4.17 |  | 3.25 | 2.00 - 5.00 |  |
| Mother’s Education: completed up to advanced level at school or higher | No | 16 | 3.18 | 2.00 - 4.33 | 0.071 | 3.38 | 1.50 - 5.00 | 0.212 |
|  | Yes | 11 | 3.70 | 2.50 - 4.25 |  | 3.00 | 2.00 - 5.00 |  |
| Father’s Education: completed up to advanced level at school or higher | No | 20 | 3.33 | 2.00 - 4.33 | 0.219 | 3.13 | 1.50 - 5.00 | 0.808 |
|  | Yes | 7 | 3.67 | 3.00 - 4.18 |  | 3.25 | 2.00 - 4.00 |  |
| Monthly income of the family (Rs.) | ≤25,000 | 10 | 3.05 | 2.00 - 3.67 | 0.057 | 3.25 | 2.00 - 5.00 | 0.976 |
|  | >25,000 | 13 | 3.67 | 2.45 - 4.33 |  | 3.25 | 2.00 - 5.00 |  |
| Ethnicity | Sinhalese | 16 | 3.48 | 2.50 - 4.18 | 0.827 | 2.88 | 1.50 - 4.00 | 0.013* |
|  | Other | 11 | 3.25 | 2.00 - 4.33 |  | 3.50 | 3.00 - 5.00 |  |
| Age at assessment | ≤12 years | 20 | 3.58 | 2.00 - 4.25 | 0.263 | 3.25 | 1.50 - 5.00 | 0.607 |
|  | >12 years | 7 | 3.00 | 2.45 - 4.33 |  | 3.00 | 2.00 - 5.00 |  |

GRB, Gender Role Behaviour; GI, Gender Indentity; CAH, Congenital Adrenal Hyperplasia

*Statistically significant at p=0.05 level
